# Home range ecology of Indian rock pythons (*Python molurus*) in Sathyamangalam and Mudumalai Tiger Reserves, Tamil Nadu, Southern India

**DOI:** 10.1038/s41598-023-36974-9

**Published:** 2023-06-16

**Authors:** C. S. Vishnu, Benjamin Michael Marshall, Chinnasamy Ramesh, Vedagiri Thirumurugan, Gautam Talukdar, Abhijit Das

**Affiliations:** 1 Wildlife Institute of India, Chandrabani, Dehradun, Uttarakhand 248002 India; 2 Department of Biological and Environmental Sciences, University of Stirling, Stirling, UK

**Keywords:** Ecology, Zoology

## Abstract

The Indian rock pythons (*Python molurus*) are classified as a near-threatened snake species by the International Union for the Conservation of Nature and Natural Resources (IUCN); they are native to the Indian subcontinent and have experienced population declines caused primarily by poaching and habitat loss. We hand-captured the 14 rock pythons from villages, agricultural lands, and core forests to examine the species' home ranges. We later released/translocated them in different kilometer ranges at the Tiger Reserves. From December 2018 to December 2020, we obtained 401 radio-telemetry locations, with an average tracking duration of (444 ± 212 days), and a mean of 29 ± SD 16 data points per individual. We quantified home ranges and measured morphometric and ecological factors (sex, body size, and location) associated with intraspecific differences in home range size. We analyzed the home ranges of rock pythons using Auto correlated Kernel Density Estimates (AKDE). AKDEs can account for the auto-correlated nature of animal movement data and mitigate against biases stemming from inconsistent tracking time lags. Home range size varied from 1.4 ha to 8.1 km^2^ and averaged 4.2 km^2^. Differences in home range sizes could not be connected to body mass. Initial indications suggest that rock python home ranges are larger than other pythons.

## Introduction

An animal's space use primarily reflects its resources' spatial availability^1^, whether those resources are shelter, food, mates, or other necessities. Home range quantification is one of the primary components in animal habitat-resource selection studies that can draw behavioral-ecological inferences across spatiotemporal scales^2^. Spatial-habitat requirements and accurate natural history information are essential for developing adaptive conservation and management plans^3^. The foraging patterns, migration and dispersal, and breeding success are all fundamental parameters directly associated with animal space use^4,5^. Home range size can correspond with the animal's body size^6,7^, where larger snakes tend to have larger ranges^8^.

Like many groups, snakes are facing a widespread decline worldwide^9^, so collecting information on home range, habitat use, and movement patterns is essential to highlight areas where human-snake confrontation needs to be reduced. Due to human activities, suitable habitat is becoming fragmented or isolated; therefore, species with low dispersal potential are highly vulnerable to local extinction^10^. A thorough understanding of spatial and temporal habitat requirements can aid resource managers in protecting species and the specific landscape features on which they depend^11^. A critical element in species conservation is knowledge concerning habitats^12,13^ as well as the movement and activity of animals within suitable habitats^14,15^. In addition, knowledge of specific resources and microhabitats used by species is vital for effective species management^16,17^.

Radio-telemetry is an essential tool for studying snakes because of snakes’ low detectability^18,19^; with radio-telemetry we can gain an insight into their requirements. The application of radio-telemetry allows for consistent determination of the snake’s location that, in turn, aids in identifying the habitat preference of snakes, frequency of its movement, distance moved, and home range^20^.

Like many snakes, the rock python’s ecology is only partially understood. Only a few studies on *P. molurus* have taken place in India; the first-ever study on the general ecology and population estimation of this species was conducted in Keoladeo National Park, Rajasthan, covering an area of 29 sq.km^21^. The *P. molurus* is a large, heavy-bodied ambush predator and non-venomous snakes with an average adult snout-vent length of six to eight metres^22^. They occupy diverse habitats in the Indian sub-continent, from dry land to wetland areas and rainforests^23^. They are semi-arboreal and prey upon small to medium-sized mammals, birds, and lizards^24^. Python activity is unimodal during winter days and bimodal in spring and summer at semi-arid conditions at early noon and late afternoons^25^. Overall, *P. molurus* populations appear to be decreasing. It has been listed in a Near Threatened category by International Union for the Conservation of Nature and Natural Resources (IUCN)^26^. Therefore, it is imperative we work to gain a better understanding of this species requirement.

In this paper, we quantify the home ranges of the *P. molurus* and explore how body size may affect variation in home range sizes.

## Methods

### Study site

**Figure 1:**
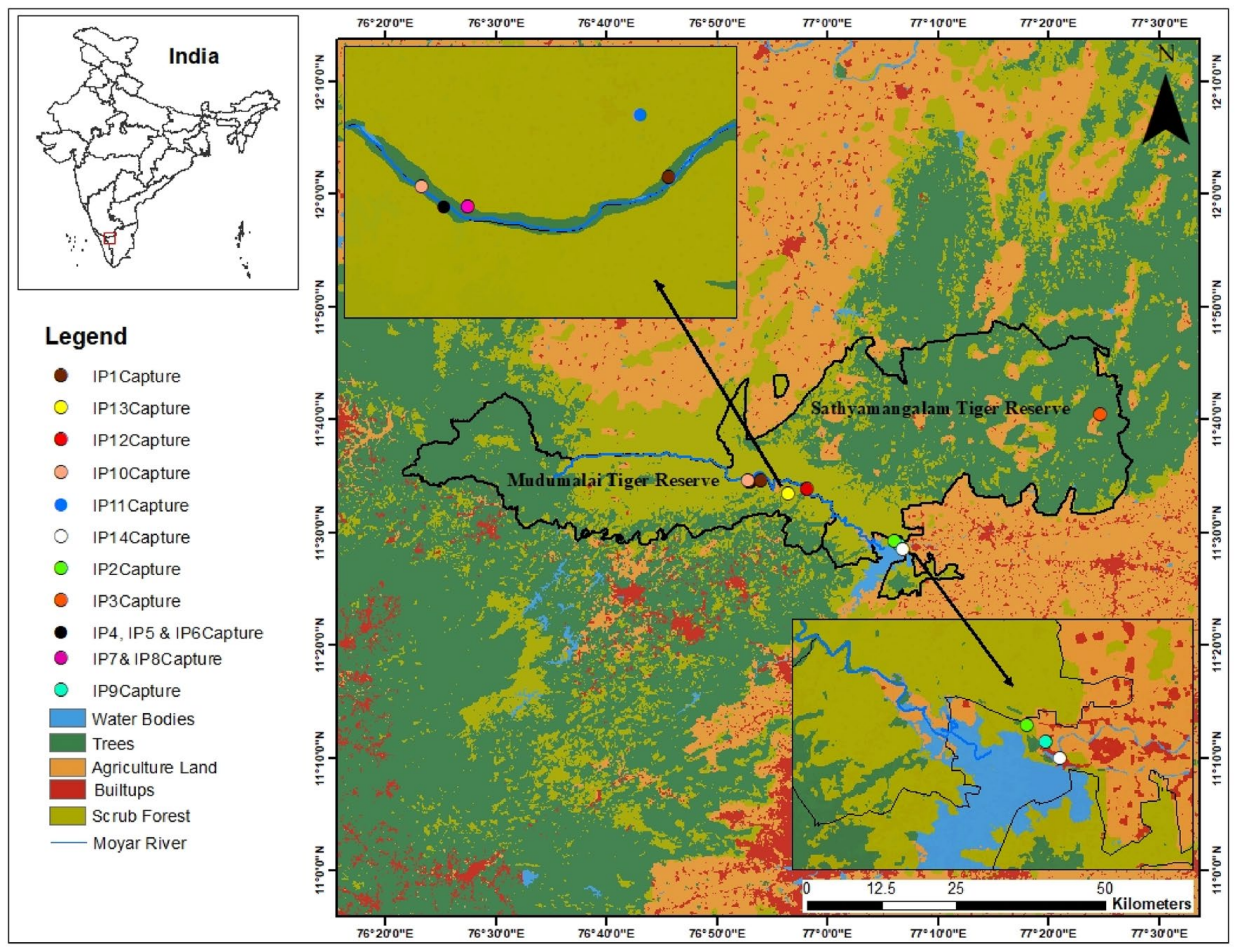
A map illustrating the capture locations of pythons in and around the Sathyamangalam and Mudumalai Tiger Reserves. Created using ArcMap 10.8.2 (Esri, Redlands, CA, USA) with Sentimental-2 (10-Meter) Land Use/Landcover Data from Esri (https://livingatlas.arcgis.com/landcoverexplorer/#mapCenter=92.202%2C26.354%2C10&mode=step&timeExtent=2017%2C2022&year=2022). Available at (https://osf.io/rhnvd).

The Moyar River is the flowing boundary between Sathyamangalam and Mudumalai Tiger Reserves (Fig. 1); the river contributes water to the Bhavani Sagar Dam in Tamil Nadu, South India. The area comes under the Nilgiri Biosphere Reserve-Sigur plateau, the connecting point of the Western and the Eastern Ghats^27^, and home to a diversity of wildlife^28,29^. The Mudumalai Tiger Reserve (MTR) is a section of the Western Ghats (11°32′–11°43′N, 76°22′–76°45′E) well-known for its rich floral and faunal diversity covering an area of 588.59 sq.km^27^. The major forest types of the reserve are Southern tropical dry thorn forest, Southern tropical dry deciduous forest, Southern tropical moist deciduous forest, Southern tropical semi-evergreen forest, moist bamboo brakes, and Riparian forests^30^. The Sathyamangalam Tiger Reserve (STR), established in 2013 and covering an area of 1400 km^2^^31^, is at the confluence of Eastern and Western Ghats^32^ and is one of the largest Tiger Reserves of Tamil Nadu (10°29′15″ to 11°43′11″ N and 76°50′46″ to 77°27′22″ E). The elevation ranges between 250 and 1450 m above sea level. The study region has three seasons: Monsoon, Post-Monsoon, and Summer^33^. It receives a mean annual rainfall of 850 mm; the mean minimum and maximum temperatures are 21 °C and 28 °C^34^.

### Capture, morphometries, and radio tracking

We located pythons by looking for tracks, shed skins, holes, termite mounds, and dense hedgerows in the forests. In addition, we rescued pythons from human settlements and agricultural areas. We used already tracked individuals to locate new individuals during the mating season. We hand-captured the pythons and temporarily restrained each individual in a snake bag within a large plastic storage container. Pythons were sedated before the surgery, measured in centimeters with a measuring tape (standard snout-to-vent length, SVL), and weighed in kilograms (kg) with a digital weighing. Also, each python was identified/marked based on the blotch pattern method^22^. Python capture and radio transmitter implantation completed following previous literature^35^. Snakes were anesthetized with isoflurane gaseous anesthesia during surgeries and implanted with VHF radio-transmitters intraperitoneally^36^ at the STR Veterinary Unit. The VHF implantable transmitters were mainly AI-2 hollohill models, consisting of 28 g and 17 g sizes. We also used the smaller ATS ARChive ARC400 Transmitter Tag model VHF implantable transmitter (14 g). Transmitters did not exceed 0.26% of the snake’s body mass.

We conducted radio tracking between December 2018 and December 2020 within Sathyamangalam and Mudumalai Tiger Reserves. We released 12 pythons in the translocated treatment 0.2–55.7 km from capture locations to areas with suitable habitats (evidenced by the presence of pythons). We released two pythons at the location where they were originally captured (control). We recorded GPS locations on each tracking day with the help of a handheld GPS device, Yagi-type antenna, and ATS receiver and used triangulation to determine the individuals’ location. We made efforts to achieve direct sightings using the homing method.

### Approval for animal use

All research activities approved by the Ministry of Environment Forests and Climate Change, Government of India, and Tamil Nadu Forest Department (No.WL5 (A)/17699/2017; Permit No. 82/2017). Our methodology is in accordance with the Ethical Principles and Guidelines for the Use of Animals for Scientific Purposes provided by the Committee for the Purpose of Control and Supervision of Experimentation on Animals (CPCSEA) on specific aspects regarding the use of animals in scientific experiments, Ministry of Environment & Forests (Animal Welfare Division) Government of India (including ARRIVE guidelines). Before surgery, isoflurane gaseous anesthesia was given to all pythons, and Chief Veterinarian performed the surgery under the supervision of the Chief Conservator Forests and Research Scientist.

### ARRIVE guidelines

The experimental procedure was performed in accordance with ARRIVE guidelines.

### Home ranges analysis and comparisons

We assessed home ranges using Autocorrelated Kernel Density Estimators (AKDE)^37^; using the ctmm package^38^. AKDEs can provide an advantage over traditional home range estimators as they account for the autocorrelated nature of animal movement data, and mitigate against biases stemming from inconsistent tracking time lags^39,40^. AKDE’s ability to handle inconsistent time lags is beneficial when tracking is conducted using manual radio-telemetry, which is vulnerable to interruptions that inclement weather, staff limitations, and equipment failures can cause.

We generated variograms to assess the pythons' range residency and determine whether we had adequate data to estimate home range areas. The variograms plotted the average square distance traveled at different time lags; we visually examined the variograms and considered those that were not reaching an asymptote as indicative of an unstable range (i.e., the home range estimates are likely to be unreliable). Range residency is a key assumption for AKDE home ranges; as such, we only use home range area estimates from individuals closest to showing range residency in further analysis (although low effective sample sizes limit confident inferences). We excluded locations for IP02, IP04, and IP07 that appeared to be homing behaviour post-translocation so the AKDEs calculated better followed the assumption of range residency.

We proceeded to fit a number of different movement models using different movement processes to the data: Ornstein–Uhlenbeck (OU), Ornstein–Uhlenbeck Foraging (OUF), and Independent Identically Distributed (IID). Ornstein–Uhlenbeck models account for the central tendency we would expect from an animal occupying a stable home range; OUF is an expansion on the OU model, additionally accounting for autocorrelation in movement speed. Lastly, IID models assume independence between locations and are the same as traditional kernel density approaches. In addition to the different processes, the range of models also considered whether the range was best described as isotropic (circular) or anisotropic (elliptical). We fit models using the perturbative hybrid residual maximum likelihood method (pHREML)^41^; and selected the best model based on dAICc values of less than two. Where possible, we recovered other characters of the home ranges—home range crossing time and effective sample sizes—however, for some individuals, the low effective sample sizes prohibited crossing time and speed estimations. To estimate the final area estimates to be reported, we opted for a weighted 95% contour and selected the point estimate for further analysis. We selected 95% over other contours as it is the most commonly used in reptile studies^42^ and adequately avoids extra-range movements^43^.

We took the 95% contours point estimates from the weighted AKDEs to assess whether the area used by pythons was related to their mass. We formulated a Bayesian regression model with a Gamma response distribution to examine whether python mass (kg) could predict area usage (km^2^): *AKDE* ~ *1* + *Mass.* We did not include any grouping effects such as sex or whether individuals had been translocated because we had insufficient numbers of each with stable home ranges. Therefore, the model was run using five individuals. We selected a weakly informative prior for the beta coefficient of mass defined by a Cauchy distribution with a location of 0.1 and a scale of 5 to keep the posterior estimation more conservative (i.e., less likely to give a false positive), given the limited sample size^44^. We ran the model over 4 chains, 6000 iterations with 1000 warmup iterations, and a thinning factor of 10. We assessed model convergence and fit using rhat values, acf plots, trace plots, posterior predictive check plot, and R^2^ values.

We used R v.4.1.1^45^; via RStudio v.2022.2.3.492^46^. We used the here v.1.0.1 package^47^ to help with relative file path definition. We used ggplot2 v.3.3.6 for creating figures^48^, with the expansions: cowplot v.1.1.1^49^, and scico v.1.3.0^50^. We used the dplyr v.1.0.9 and stringr v.1.4.0 packages for data manipulation^51,52^. We used sp v.1.4.7^53^ for manipulation of spatial data and the ctmm v.0.6.1^38^ to estimate space use. We used brms v.2.17.0^54–56^ to run Bayesian regression models, with bayesplot v.1.9.0^57,58^, performance v.0.9.1^59^, and tidybayes v.3.0.2^60^ to assess model outputs. We generated R package citations with the aid of grateful v.0.0.3^61^. The code we used was based upon that used by^62^ that is available at https://osf.io/rxu6f/.

## Results

### Body size and tracking

We tracked 14 individuals, eight males and six females ranging in mass from 3.3 kg to 36.25 kg (mean 10.65 ± SD 10.75 kg) and snout-to-vent length from 1.72 m to 3.76 m (mean 2.55 ± SD 0.62 m). Twelve of the pythons were translocated (Table 1).

**Table 1:** Characteristics of the tracked pythons.

ID	Sex	Resident translocated	Release range	Mass	Length	SVL	Tail length
IP01	Female	Resident	0	32	412	376	36
IP02	Male	Relocated	22.5	12.75	280	248	32
IP03	Female	Relocated	55.7	11.05	350.5	310.3	40.2
IP04	Female	Relocated	1.9	36.25	370	342	28
IP05	Male	Relocated	3.2	11.35	263	229	34
IP06	Male	Relocated	3.3	11.3	272	252	20
IP07	Male	Relocated	4.7	5.1	222	194.5	27.5
IP08	Male	Relocated	3.13	3.3	198	172	26
IP09	Male	Relocated	21.2	10.2	286	251	35
IP10	Male	Relocated	9.93	10.15	268	238	30
IP11	Female	Relocated	2.32	6.65	226	197.5	28.5
IP12	Female	Relocated	0.2	31.4	384	340	44
IP13	Male	Resident	0	12.8	237	211	26
IP14	Female	Relocated	10.96	24.8	270	215	55

Release range (km), mass during first capture (kg), length is SVL + Tail length (cm), SVL = Snout-vent-length (cm), Tail length (cm).

While aiming for a tracking time lag of 24 h, the resulting mean time lag was 16 ± SD 35 days (range 0.2–238 days; Supplementary Fig. 1), and the median was 5 days. The tracking duration ranged from 44 to 695 days with a mean of 444 ± 212 days; during this time, a mean of 29 ± SD 16 data points collected per individual (range 8–65; Table 2).

**Table 2:** Tracking summary.

ID	Start date	End date	Duration (days)	Mean ± SD time lag (days)	Number of datapoints	95% AKDE point estimate (km^2^)	Lower 95% CI of AKDE estimate (km^2^)	Upper 95% CI of AKDE estimate (km^2^)	Top fitting movement
IP01	2018-12-16	2020-11-10	695.1	10.9 ± 29.9	65	1.51	0.97	2.17	OU anisotropic
IP02	2019-01-20	2020-11-10	660.3	17.8 ± 32.3	38	–	–	–	–
IP03	2019-01-25	2019-03-19	53.2	5.3 ± 6	11	–	–	–	–
IP04	2019-01-21	2020-09-08	595.9	21.3 ± 41.6	29	4.48	2.55	6.96	OU anisotropic
IP05	2019-01-21	2020-02-22	397	11.3 ± 17.2	36	8.07	5.8	10.7	IID anisotropic
IP06	2019-01-07	2020-10-27	659	12 ± 25.5	56	1.59	1.2	2.03	IID anisotropic
IP07	2019-03-07	2020-03-12	370.9	24.7 ± 38.8	16	–	–	–	–
IP08	2019-03-08	2020-12-04	637.1	30.3 ± 49.1	22	6.03	3.08	9.96	OUf anisotropic
IP09	2019-05-19	2019-07-02	44.1	6.3 ± 11.3	8	–	–	–	–
IP10	2019-05-29	2020-10-30	520.2	22.6 ± 48.9	24	–	–	–	–
IP11	2019-07-27	2020-11-06	467.9	14.6 ± 31.7	33	–	–	–	–
IP12	2019-07-26	2020-11-30	493.1	22.4 ± 43.7	23	–	–	–	–
IP13	2019-12-13	2020-11-02	325.1	19.1 ± 42.1	18	–	–	–	–
IP14	2019-12-26	2020-10-12	290.8	13.8 ± 51.4	22	–	–	–	–

### Home ranges analysis and comparisons

**Figure 2:**
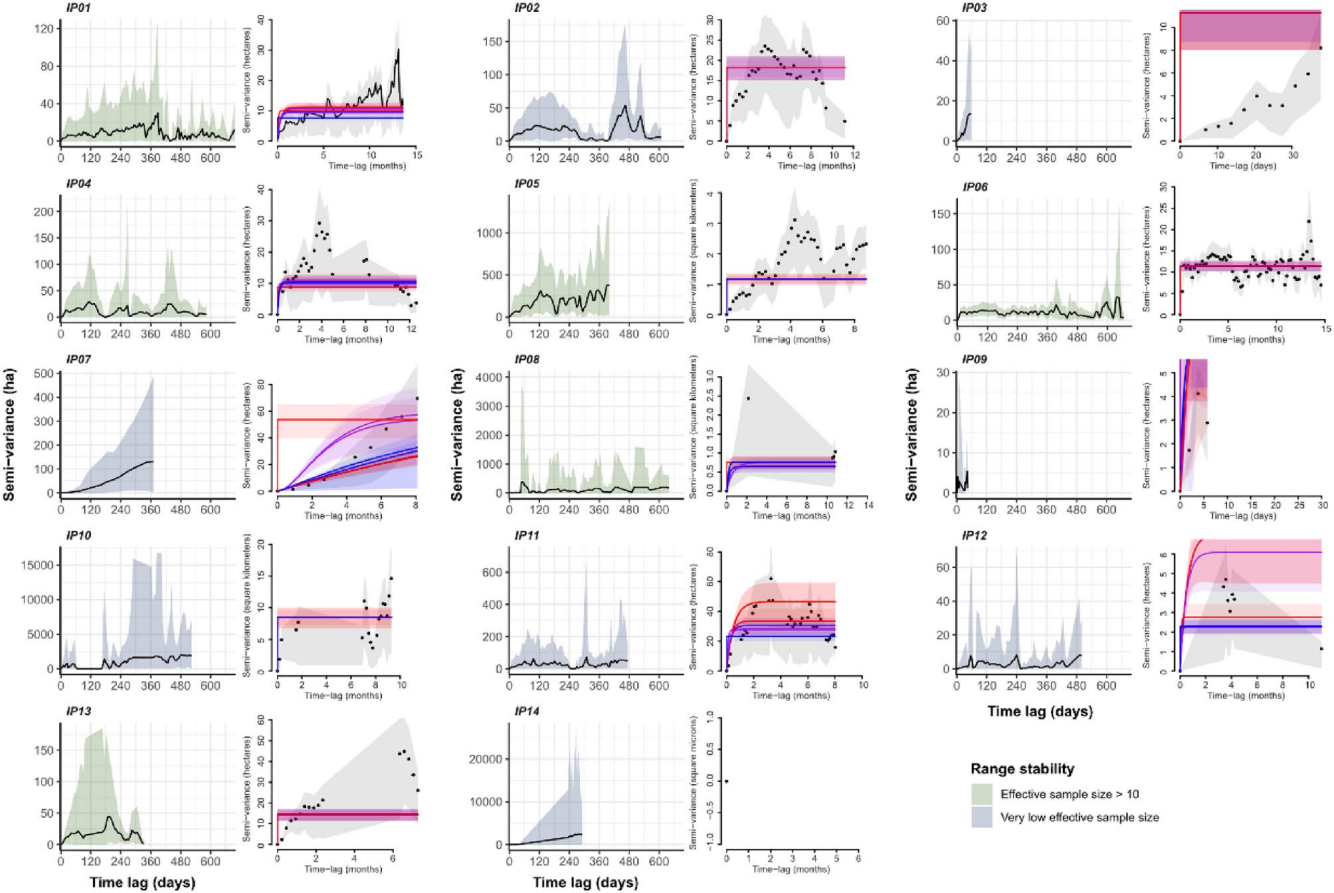
Variograms generated for all individuals with highlighting to show those with sufficient effective sample sizes to be included in further analysis. Right hand plots contain zoomed views to show the movement model fits.

Our initial examinations of the variograms suggested questionable range stability; therefore, home ranges should be interpreted with caution (Fig. 2). Some of the individuals tracking data yielded extremely low effective sample sizes, undermining our confidence in any resulting estimates of area (IP10, 11, and 12). Overall, python tracking data yielded a mean effective sample size of 13.0 ± SD 17.2 (0.02–56.0). When limited to individuals with effective sample sizes over ten, the mean effective sample size is 29.6 ± SD18.7 (range 11.6–56.0). From the potentially more-stable individuals, we were able to retrieve three estimates of home range crossing time, with a mean of 6.0 ± SD 3.9 days (range 2.7–10.3 days).

**Figure 3:**
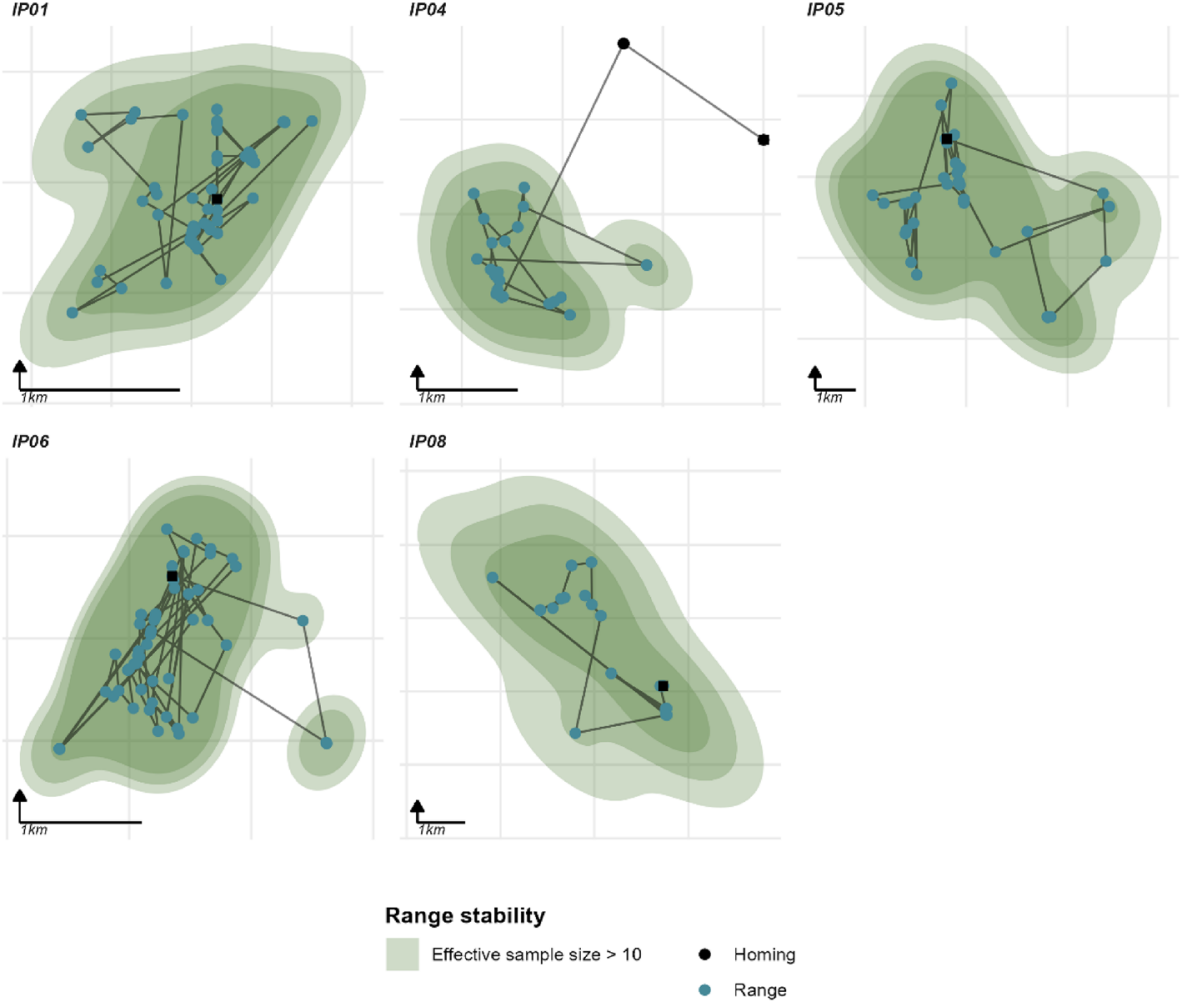
The range resident individuals AKDE areas mapped. Square points indicate the release site of the individual. Differing levels of opacity show the 95% confidence intervals surrounding the 95% contour estimate. Note all individuals are all on independent scales.

**Figure 4:**
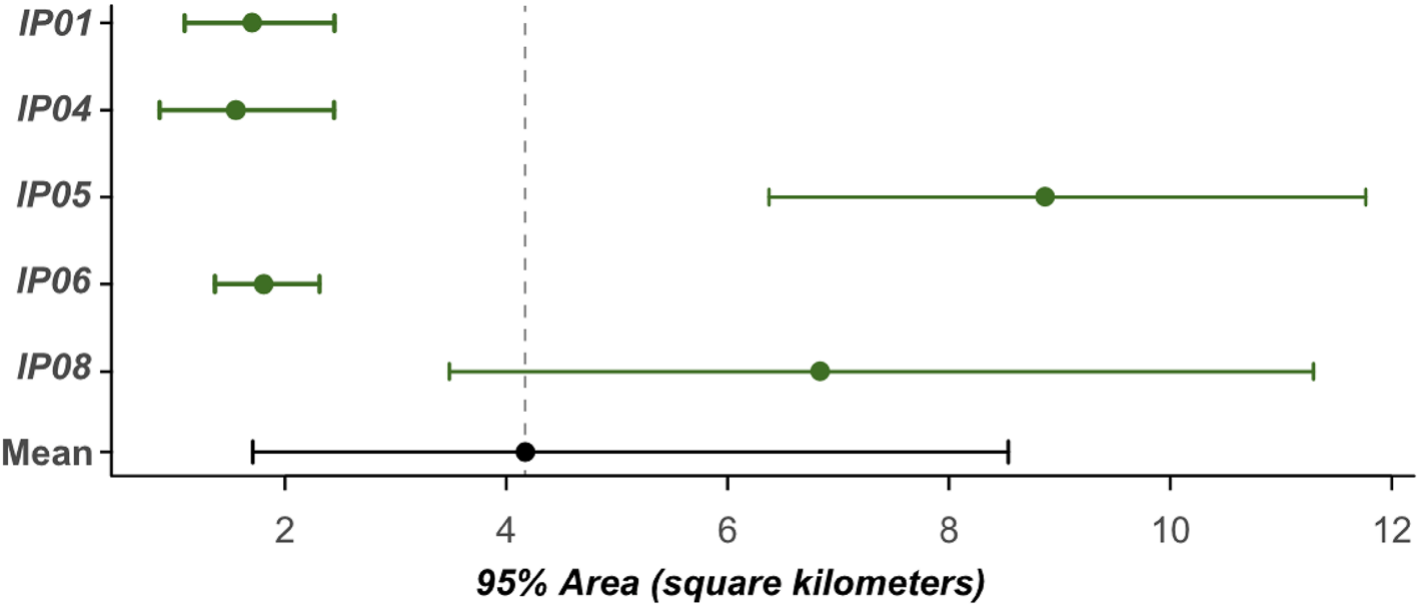
The estimated home range sizes of rock pythons, alongside the mean home range size. All estimates are paired with 95% confidence intervals.

We estimated the weighted AKDE home ranges for all individuals but only used those with effective sample sizes above 10 for further analysis. The 95% contour point estimates had a mean of 3.7 ± SD 1.4 km^2^ (range 1.36–8.1 km^2^, n = 5; Fig. 3). But this hides some of the variations; the lowest area estimate (lower 95% confidence interval) was 0.8 ha and highest was 10.7 km^2^. Using ctmm’s meta function (that takes into account uncertainty) on the top fitting models the population mean was 4.2 km^2^ (95% CI. 1.7–8.5 km^2^, n = 5; Fig. 4). Although the sample size prohibits robust statistical comparisons, females appear to have smaller ranges 1.6 km^2^ (95% CI. 1.2–2.2 km^2^, n = 2) compared to males 5.0 km^2^ (95% CI. 4.1–6.0 km^2^, n = 3). Of the stable individuals only one was resident (IP01), and had a range of 1.7 km (95% CI. 1.1–2.4 km^2^). The translocated individuals had a larger mean range of 4.8 km^2^ (95% CI. 1.7–10.9 km^2^, n = 4).

IP01 and IP04’s movements were best described by an OU anisotropic model suggesting the presence of central tendency (although IP04’s dAIC was within 2 of the OUf model). IP08 also supports this, but the OUf anisotropic performs best, showing that IP08’s data also contains autocorrelated velocities (although IP08’s dAIC was within 2 of the OU model). IP06’s movements were best described using the IID anisotropic model, suggesting that location data could be modelled as independent. The anisotropic or elliptical nature of the home ranges is visible in Fig. 3.

**Figure 5:**
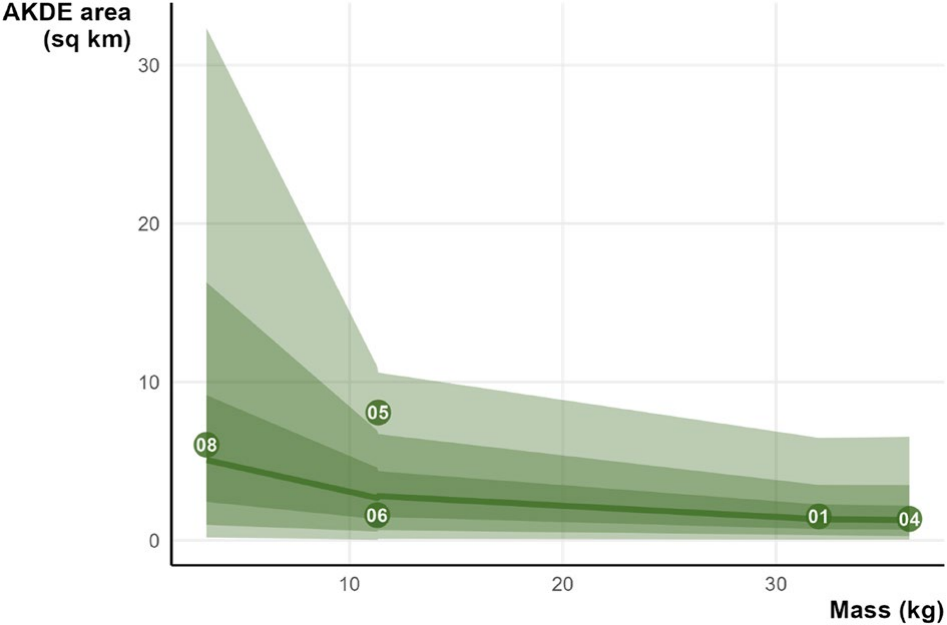
Predicted draws from the 95% contour area point estimate ~ mass model, illustrated with original data points (with python IDs) and 95, 80, and 50% credible intervals.

Our Bayesian regression model successfully converged, confirmed by rhat values ~ 1 and diagnostic plots (that can be found at: https://osf.io/d58rs/). The resulting model suggested a negligible negative correlation between 95% home range area and mass (*β* = 0.02, 95% CI 0.00–0.05; Fig. 5). However, the estimate was very uncertain. The posterior predictive check plot revealed that predicted draws did not fit particularly well with the original data. The R^2^ value of 0.36 was paired with very wide credible intervals (95% CrI 1.23e-07, 0.58).

## Discussion

In our study, the rock python from the Moyar Valley, Southern India, occupied an average home range of 3.7 km^2^ (AKDE 95% point estimate) or 4.2km^2^ using ctmm meta-analysis functionality. While inconsistencies in the manual tracking affected the home range estimation for all the individuals, five individuals provided high enough effective sample sizes to begin to gauge home range size (Fig. 2).Homing has impacted home range estimates in a few individuals. Even though IP02, IP04, and IP07 had stable ranges, we excluded their home range estimations due to their homing behaviour after translocation. If we included these individuals in the home range estimation, the AKDEs would estimate the entire range after translocation as their resident ranges. By excluding them from the home range estimation, we have reduced the biases.

The five individuals range estimates come with broad confidence intervals highlighting the uncertainty surrounding our estimates, with the point estimates alone ranging by orders of magnitude. The largest range (with an effective sample size > 10) was from IP7, a translocated male, who ranged approximately 21.7 km^2^. The only resident python with a more stable range appeared to have one of the smaller home ranges at 1.5 km^2^; however, translocated individuals could also exhibit smaller ranges (e.g., IP02 & IP04 covering ranges of ~ 1.4 km^2^). While our estimates offer insight into rock python home range size, a larger sample with longer tracking periods would help reduce the uncertainty; and tracking pythons in different sites will help build a broader picture of their movements.

We did not see any clear evidence that larger individuals had comparatively larger home ranges (although the model fit was poor). This contrasts to clearer differences in other similarly sized species. For example, larger King Cobras (*Ophiophagus hannah*) in Northeast Thailand had larger home ranges (calculated using Kernel density estimators)^63^, suggesting a potential connection between size, metabolic demands, and foraging effort. These differences may be linked to the foraging mode. Large-bodied pythons prefer large prey items like ungulates, as ambush foraging snakes have comparatively larger prey sizes than active foraging species; this can curtail their movements due to the time required to digest heavy meals^64^.

While we could not test the differences statistically (due to low sample size and misbalance in the translocation history of individuals), females (IP01 resident and IP04 translocated) appeared to have smaller ranges. This contrasts with other snakes, such as White-lipped Pit Vipers (*Trimeresurus albolabris*); the frequency of movements and distances moved by female snakes are significantly higher than the males in Southern China^65^. However, in a study on other large snakes, Eastern Indigo Snakes (*Drymarchon couperi*); female home ranges were 0.76 km^2^, while male home ranges averaged 2.02 km^2^, estimated using Kernel density estimates, respectively^66^. It is suggested that larger male ranges are likely a result of mate-searching behaviour^67^. In Diamond Pythons*, Morelia spilota spilota* males moved long distances, often daily, compared to females^68^. Such increased movement during a portion of the year (the mating season) could be the driver behind the larger overall ranges. High female movements tend to be connected to oviposition^69^. We did not witness nesting behaviors in any of the females; presumably for IP04, the effect of translocation hindered nesting activity.

Studies on the space use of a sister species, Burmese Python *Python bivittatus* have been done in different regions: native (Asia) and introduced ranges (North America). The (Minimum Convex Polygon) home ranges of invasive *P. bivittatus* , were reported to average 22.5 Sq.km^70^, whereas in their native range^71^ the average home range is 0.12 km^2^ (although only over 24 days). A longer duration study on native *P. bivittatus* movements indicates that Goodyear’s estimates^71^ are likely underestimates of python space requirements. The study^72^ did not calculate home ranges, instead reporting dynamic Brownian Bridge Movement Model 99% confidence areas that approximately average 0.99 km^2^. As dynamic Brownian Bridge Movement Models aim to quantify uncertainty surrounding a movement path rather than a home range^73^, we can expect the range to be larger than 0.99 km^2^. It would appear that the rock python’s home range is likely to be considerably larger than the *P. bivittatus* (although more robust comparisons would require recalculation of ranges using AKDEs). Previous studies on snakes, including pythons, have shown that the habitat use and their movements can differ in relation to many factors such as body size^74,75^, sex^75,76^, season^75,77–80^, and prey availability^81^. Studies focusing particularly on large constrictors, have observed the shift of habitat uses seasonally^68,82,83^, movement in response to weather changes, and home range size differing between native and translocated individuals^84,85^. Failure to track snakes for a long enough duration to capture seasonal variation, or season specific behaviour (e.g., mate searching, that here may have been disrupted by translocation), may explain inconsistencies in range estimates and relationships between home range and individual constrictor’s characteristics.

VHF telemetry remains the primary option for determining snake home range study because of the snake’s restrictive body plan. The human or aircraft resources required for VHF tracking increase rapidly with tracking frequency, making data collection expensive and infeasible for high-frequency sampling schedules^86^. Our study was limited to diurnal hours because of the Tiger Reserve rules. Various obstacles affected the tracking of pythons, such as the small workforce, limited funding, and an inaccessible landscape with high densities of wild hazards, especially elephants and thorny bushes of *Prosopis* and *Carissa*. The Covid-19 Pandemic compounded the difficulties with data collection. This study resulting low tracking frequency will miss small-scale movements, potentially impacting the home ranges estimation^87^. Therefore, we have emphasized the findings from individuals with an effective sample size over 10 using an analysis method that helps to mitigate the biases of low, inconsistent tracking data^88^. Fortunately, wide-time intervals between location data collection may be less of a concern for large slow-moving animals^89^. This may apply in the case of pythons as they are slow-moving animals incapable of exceeding 1.6 kph^90^, and with a predominantly ambush-focused foraging style^91,92^.

To conclude, our home range estimate provides baseline information on the *P. molurus* that is a key early step in exploring what influences rock python movement and space requirements. The highest home ranges observed in other species of python: Carpet Pythons (*Morelia spilota*) had home ranges from 0.18 Sq.km^82^ to 0.23 Sq.km^93^, and diamond pythons (*Morelia spilota spilota*) had home ranges of 0.27 km^2^ for females and 0.52 km^2^ for males^68^, would initially suggest rock pythons require larger areas. However, again, confidence in any comparisons must be tempered as the home range estimation method differ between the studies and the predominance of translocation individuals in our study. Further work is required to identify the drivers behind home range size and initial investigations into how pythons select home range areas to improve these findings' relevance to conservation planning.

In a case study conducted in the Jalpaiguri District, West Bengal, it was observed that the *P. molurus* often inhabits roads, railway tracks, and human-dominant areas. This increased proximity to human settlements is a result of the destruction of forests and the escalating human activity in forest fringe areas. As a consequence, the potential for human-wildlife conflict is significantly high, as reported in a study^94^. In this context, home range estimates can assist in mitigating conflicts between humans and  pythons. For species that overlap with human activities, such as agricultural or urban areas, knowledge of their home range can inform land-use planning and management decisions. By identifying areas of potential conflict and implementing appropriate measures such as wildlife corridors and buffer zones, managers can reduce negative interactions and promote coexistence.

When translocating or reintroducing these pythons, home range estimates help determine suitable release sites and ensure that the new habitat provides sufficient resources and space for the species. This information increases the success rate of translocation and reintroduction efforts, enhancing the chances of population establishment and persistence.

## Supplementary Information

Supplementary Figure 1.

## Data Availability

The datasets used and/or analysed during the current study are available from the corresponding author upon reasonable request.
