# Supplementary material for: Home range ecology of Indian rock pythons (Python molurus) in Sathyamangalam and Mudumalai Tiger Reserves, Tamil Nadu, Southern India
Source: Sci Rep. 2023 Jun 16;13:9749. doi: 10.1038/s41598-023-36974-9 (PMC10275859; doi:10.1038/s41598-023-36974-9)
Supplement: Supplementary file 1 — Supplementary Figure 1. [file 41598_2023_36974_MOESM1_ESM.pdf]

Supplementary Material

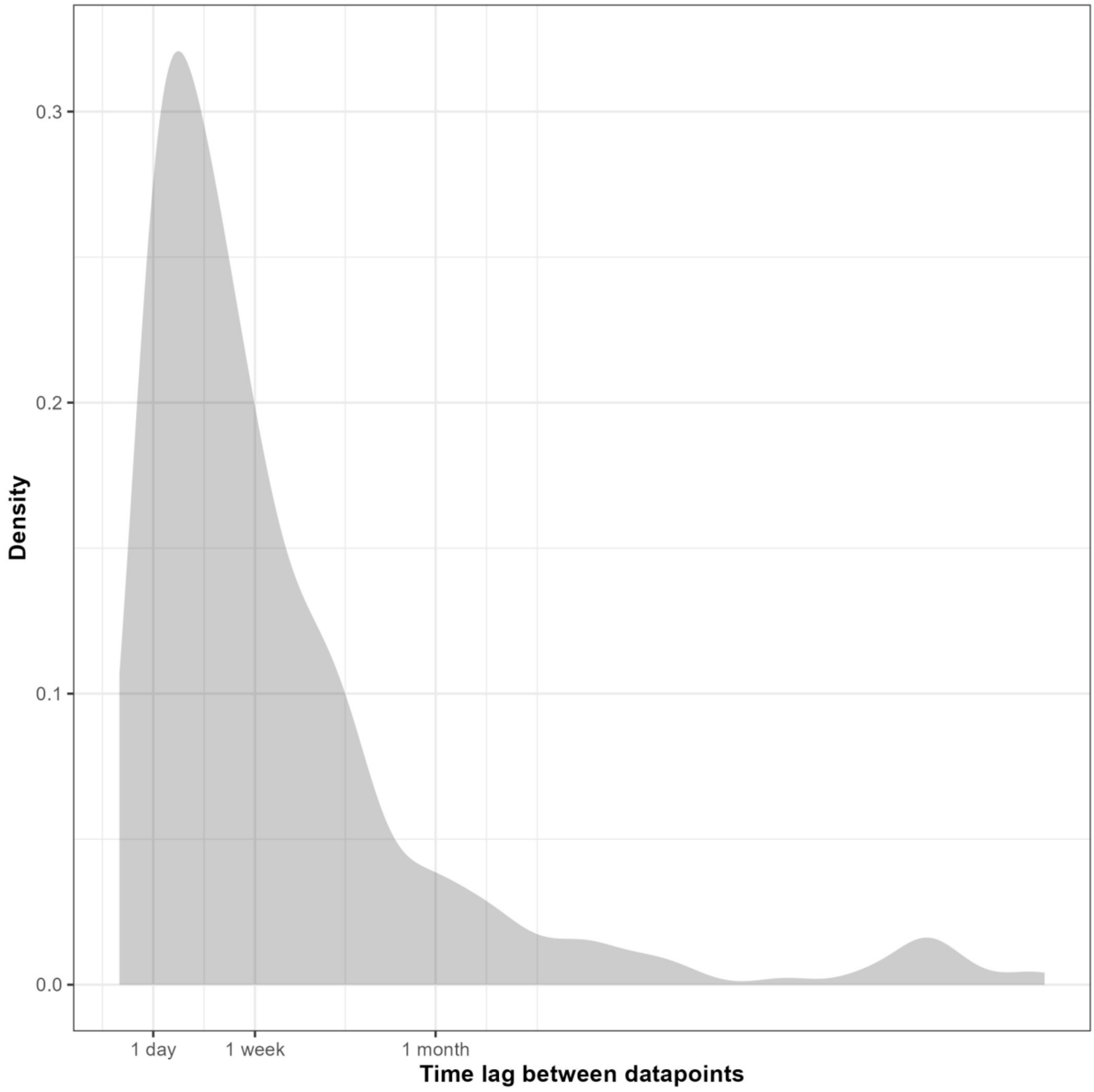

Supplementary Fig .1 Distribution of the time lags between subsequent data points. Note that the x scale is square-rooted
